# *Ehrlichia chaffeensis* proteomic profiling reveals distinct expression patterns of infectious and replicating forms

**DOI:** 10.3389/fcimb.2025.1463479

**Published:** 2025-04-14

**Authors:** Chandramouli Kondethimmanahalli, Roman R. Ganta

**Affiliations:** ^1^ Center of Excellence for Vector-Borne Diseases, Department of Diagnostic Medicine/Pathobiology, College of Veterinary Medicine, Kansas State University, Manhattan, KS, United States; ^2^ Department of Veterinary Pathobiology, Bond Life Sciences Center, College of Veterinary Medicine, University of Missouri, Columbia, MO, United States

**Keywords:** human monocytic ehrlichiosis (HME), *Ehrlichia chaffeensis*, rickettsiales, tick-borne diseases, intracellular bacteria, infectious and replicating forms, proteomics

## Abstract

*Ehrlichia chaffeensis* is a tick-transmitted rickettsial pathogen responsible for causing human monocytic ehrlichiosis (HME). The pathogen’s developmental cycle includes infectious dense-core cells (DCs) and non-infectious replicating cells (RCs). Defining the proteins crucial for the two growth forms is of fundamental importance in understanding the infection and replication process, which also aids in identifying novel therapeutic targets against HME and other related rickettsial diseases. *E*. *chaffeensis* organisms cultivated in a macrophage cell line were purified as DC and RC fractions and subjected to comprehensive quantitative proteome analysis. From triplicate sample analysis, we identified 195 proteins as commonly expressed in both the DC and RC forms, while an additional 189 proteins were recognized as exclusively expressed in the RC form. Equal numbers of commonly expressed proteins in the RC and DC forms and having substantially more proteins exclusively expressed in the metabolically active RC form may reflect specific functional priorities of *E. chaffeensis* supporting its replication within a phagosome. The high abundance of metabolic processes and transport proteins in the RC compared to the DC form may reflect its higher metabolic requirements and interactions with a host cell supporting its intraphagosomal replication. This study provides comprehensive proteome data for *E. chaffeensis* which will be valuable for a better understanding of protein expression dynamics during its infectious and replicating stages.

## Introduction

*Ehrlichia chaffeensis* is one of the most common tick-transmitted intracellular rickettsial pathogens in humans and vertebrate hosts (Dumler et al., 1993; Walker and Dumler, 1996; Davidson et al., 2001; Paddock and Childs, 2003). *E*. *chaffeensis* infection is responsible for human monocytic ehrlichiosis (HME) and it can progress to a fatal outcome in the elderly, children, and immune-compromised individuals (Paddock and Childs, 2003; Walker and Dumler, 1996). It is primarily harbored by *Amblyomma americanum*, which is an aggressive tick widely distributed in the southeastern, eastern, and parts of the midwestern regions of the USA (Childs and Paddock, 2003). While *E. chaffeensis* infections are primarily attributed to tick transmission, people receiving blood transfusions and organ transplantations are also at high risk of acquiring infections (McQuiston et al., 2000; Sachdev et al., 2014). The pathogen primarily undergoes a biphasic life cycle, which includes a small dense-core cell (DC) form, which is the infectious form and the larger replicating form, regarded as the reticulate cell (RC) form (Dedonder et al., 2012 and Eedunuri et al., 2018). We previously reported that the two distinct morphological forms in tick cells and macrophage cultures exhibit notable morphological variations (Dedonder et al., 2012). The pathogen progression in the host cells begins with the attachment and internalization of DCs to the host cell membrane followed by their engulfment, replication, and transformation into the non-infectious RCs within a morulae (Dedonder et al., 2012; Yu et al., 2000). RCs mature to DCs and then release by exocytosis or by complete lysis of infected host cells to initiate another cycle of infection and replication (Dedonder et al., 2012). These two stages of *E. chaffeensis* likely result in proteomic reorganizations to support the biphasic lifecycle (Fisher et al., 2015).

Prior proteomic studies related to *E. chaffeensis* that assess the total proteome, membrane proteome, immunogenic proteome, and Ehrlichia-containing phagosome proteomes have been reported (Singu et al., 2006; Seo et al., 2008; Lin et al., 2011; Zhu et al., 2009; Wakeel et al., 2011; Luo and McBride, 2012; Kondethimmanahalli and Ganta, 2022). However, proteins uniquely and commonly expressed in the two distinct forms (DC and RC) of *E*. *chaffeensis* remain to be defined. Defining the differentially expressed proteins for the establishment of infection and replication is of fundamental importance and such data will be valuable for discovering novel diagnostic and therapeutic targets, and promoting the goals of subunit vaccine development against HME and other related rickettsial diseases. In our recent studies, we reported transcriptome and proteome differences in purified *E*. *chaffeensis* wildtype and mutant bacteria by taking advantage of the RNA deep sequencing, two-dimensional electrophoresis (2DE), and iTRAQ-based shotgun proteome deep sequencing methods; we assessed the pathogen’s global protein expression differences resulting from functional gene disruptions during the pathogen’s attenuation growth *in vivo* (Kondethimmanahalli and Ganta, 2018; Kondethimmanahalli et al., 2019).

In this study, *E*. *chaffeensis* organisms cultured in a macrophage cell line were purified and fractionated as DC and RC forms by renografin density gradient centrifugation and subjected to global proteome analysis. We then cataloged and quantified the proteins expressed primarily in the two distinct replicating (RC) and infectious (DC) forms of *E. chaffeensis*.

## Materials and methods

### Infection and purification of *E. chaffeensis* from infected macrophages and fractionation of DCs and RCs

*E*. *chaffeensis* was grown in the canine macrophage cell line, DH82, as described previously (Cheng and Ganta, 2010) and processed for use in total protein isolation from DC and RC purified fractions as we previously described (Kondethimmanahalli and Ganta, 2022; Kondethimmanahalli et al., 2019). Briefly, *E*. *chaffeensis*, grown in six T-150 flasks with a confluent monolayer of DH82 cells with infection reaching 80%–90%, were harvested and centrifuged at 500 × g for 5 min at 4°C. The supernatant was collected in 50 ml tubes and kept on ice. The cell pellets were resuspended in 1× phosphate-buffered saline (PBS) containing protease inhibitors cocktails (Roche, Indianapolis, IN) and homogenized on ice by passing through a 23 g needle in a 10 mL syringe. The disrupted cell suspension was centrifuged at 500 × g for 5 min at 4°C and the supernatant containing *E. chaffeensis* organisms was mixed with the supernatant in 50 ml tubes and filtered through a 2.7 μm membrane filter (Millipore, Billerica, MA). The filtrate was then centrifuged at 15,000 × g for 15 min and the pellet was suspended in 1 ml of PBS. The bacterial suspension was overlaid on top of a step gradient solution containing 35%, 25%, and 15% renografin in PBS and centrifuged at 100,000 × g for 1 h at 4°C using a swinging bucket rotor (S50-ST) in a Sorvall MTX150 bench-top ultracentrifuge (Waltham, MA). DC and RC fractions located at the interfaces of 25% and 35% renografin were collected and diluted with 3 volumes of PBS, and then centrifuged at 15,000 × g for 15 min at 4°C to recover the bacterial pellets. The purified DC and RC pellets were washed once with PBS to remove residual renografin and the centrifugation step was repeated to recover final purified bacteria and used for protein extractions.

### Protein extraction, sodium dodecyl sulfate–polyacrylamide gel electrophoresis, and Western blot analysis

DC and RC pellets of bacteria were resuspended in lysis buffer containing 8M urea, 2M thiourea, 4% CHAPS, and protease inhibitors. Samples were sonicated on ice for 30 s using a sonic dismembrator (Fisher Scientific, Hampton, NH) and centrifuged at 15,000x g for 15min at 4°C. Proteins were precipitated and purified using a Readyprep 2D cleanup kit (BioRad, Hercules, CA) and then quantified using a Bradford protein assay kit (BioRad). Furthermore, 20 μg of protein derived from DC, RC, or uninfected DH82 cells was suspended in SDS sample buffer (Invitrogen, Carlsbad, CA) and then the samples were boiled for 5 min and subjected to protein separation in a Mini-PROTEAN Precast TGX 4% to 15% polyacrylamide gel (Bio-Rad) at 100 V for 90 min. The gels were separated from the cast and stained using a Novex Colloidal Blue stain kit (Invitrogen). For the detection of beta-actin protein in uninfected DH82 cells, the resolved proteins were transferred onto a 0.45-micron nitrocellulose membrane (Thermo Fisher Scientific, Rockford, IL), probed with β-Actin (13E5) rabbit monoclonal antibody (Cell Signaling, Beverly, MA), then with a secondary anti-rabbit antibody conjugated with horseradish peroxidase (Sigma-Aldrich, St. Louis, MO, USA), and finally ECL Western blotting detection reagents (Amersham, Buckinghamshire, UK) were used for the signal detection.

### LC-MS/MS analysis of DC and RC proteins

In total, 200 μg of cell-free DC and RC proteins were reduced with 5 mM Tris (2-carboxyethyl) phosphine and alkylated using 10 mM iodoacetamide and diluted 7 times with 100 mM ammonium bicarbonate. Proteins were subsequently digested with trypsin 1: 50 enzyme-protein ratio (Promega, Madison, WI) and Endoproteinase Lys-C 37°C as described (Wohlschlegel, 2009; Florens et al., 2006). Digestions were stopped with the addition of 5% formic acid and then desalted using Pierce C18 tips (Thermo Fisher Scientific) and vacuum dried. Peptides were fractionated using a 25 cm long, 75 uM inner diameter fused silica capillary column packed in-house with bulk C18 reversed phase resin in buffer A (water with 3% DMSO and 0.1% formic acid) and buffer B (acetonitrile with 3% DMSO and 0.1% formic acid). A 140 min increasing gradient of 5% to 80% acetonitrile was delivered using a Dionex Ultimate 3000 UHPLC system (Thermo Fisher Scientific) at a flow rate of 300 nl/min. Peptides were ionized using a distal 2.2 kV spray voltage and a capillary temperature of 275°C and electrosprayed into an Orbitrap Fusion Lumos mass spectrometer (Thermo Fisher Scientific) where fragment ions were analyzed by tandem mass spectrometry (MS/MS). Data was acquired using a Data-Dependent Acquisition (DDA) strategy comprised of a full MS1 scan at 120,000 resolutions followed by sequential MS2 scans (Resolutions = 15,000) with 3-second cycle times. Data analysis was performed using the Max Quant and Perseus software packages (Cox and Mann, 2008). Peptide and protein identifications were generated by the Andromeda search engine after searching against *E. chaffeensis* protein databases (Taxonomy ID 943, GenBank Accessions no. CP000236). Peptide and protein quantitations were determined by MS1-based quantitation of chromatographic peak areas. Maxquant intensity data were imported into the Perseus (Tyanova et al., 2016) algorithm which was used to impute missing values and determine which proteins were differentially abundant across samples using a two-tailed t-test.

### Western blot by 1-D gel analysis

In total, 20 μg each of DC and RC proteins were separated on polyacrylamide gels transferred onto nitrocellulose membrane (Thermo Fisher Scientific, Rockford, IL). *E*. *chaffeensis* p28‐Omp 19 specific and total immunogenic proteins were assessed using p28 monoclonal or with *E. chaffeensis* polyclonal sera. Corresponding secondary antibodies conjugated with horseradish peroxidase (Sigma-Aldrich, St. Louis, MO, USA) were used. ECL Western blotting detection reagents *(*Amersham, Buckinghamshire, UK) were used for the signal detection.

## Results

### Proteome of the *E. chaffeensis* DC and RC forms

**Figure 1:**
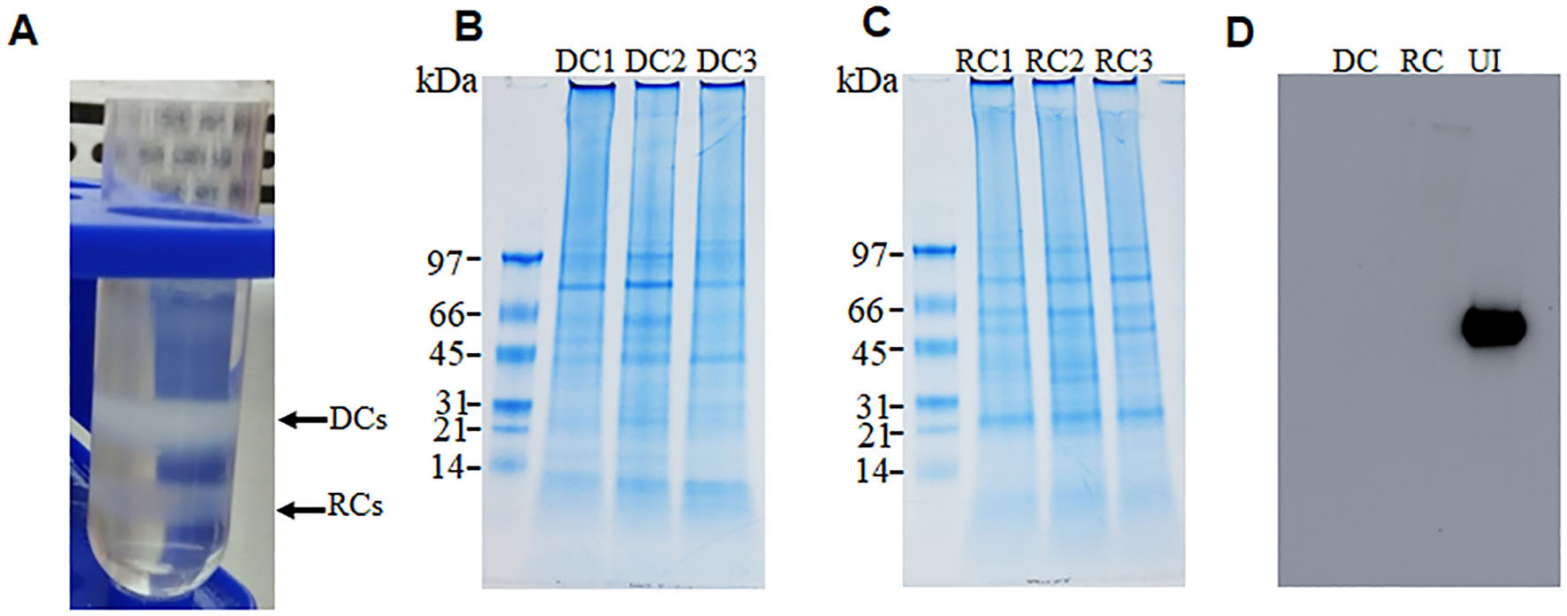
Purification of dense-core cells (DCs) and reticulate cells (RCs) of *E*. *chaffeensis.* Two growth forms were purified by renografin density gradient centrifugation **(A)**. Replicate fractions of *E. chaffeensis* were lysed in lysis buffer and sonicated on ice for 30 s and then centrifuged at 15,000x g for 15min. Proteins were precipitated and desalted for SDS PAGE analysis. Further, 20 ug of protein was run on 4%–15% polyacrylamide gels and then stained with colloidal blue stain **(B, C)** or transferred to nitrocellulose membranes for Western blot analysis. Membranes were probed with monoclonal anti-beta-actin antibody 13E5 followed by horseradish peroxidase-conjugated anti-rabbit IgG antibodies and the signal was detected using ECL reagents **(D)**.

*E*. *chaffeensis* organisms cultured *in vitro* in the canine macrophage cell line (DH82) were purified as DC and RC fractions by density gradient centrifugation (**Figure 1A**). Total proteins recovered from three replicates of DC and RC forms were resolved on a polyacrylamide gel electrophoresis (PAGE) (**Figures 1B, C**) and the purity of the proteins was confirmed by the lack of the host cell-specific beta-actin, assessed by Western blot analysis using the actin-specific antibody (**Figure 1D**). Comprehensive proteome analysis using high-resolution LC-MS/MS was performed to identify and quantify protein profiles for three replicates from DC and RC fractions. The protein expression profiles were then compared between the RC and DC forms for the three replicate samples (**Supplementary Figures S1A, B**). The scatter plots of the replicate samples of DC and RC revealed a high degree of correlation among replicates (R², >0.97 and >0.94, respectively), whereas the correlation was the lowest when comparing proteins identified in the DC with those in the RC form (R²= 0.148) (**Supplementary Figure S1C**).

**Figure 2:**
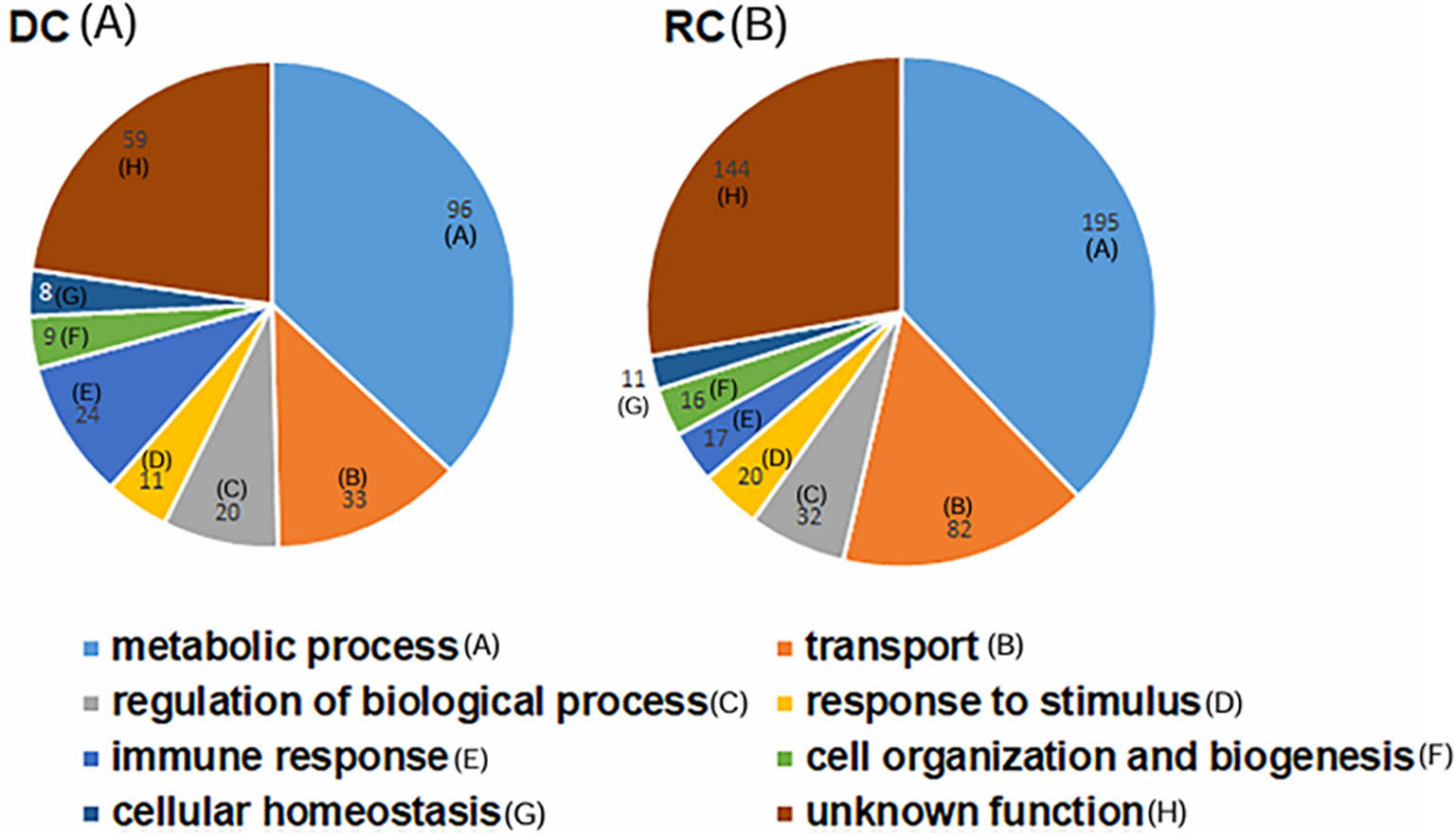
Pie charts representing functional assignment distribution of the identified proteins into different categories of biological processes. DC **(A)** and RC **(B)** proteins of *E. chaffeensis.* Proteins in the DC form had decreased abundance compared with the RC form **(B)**. The number of proteins belonging to each biological process is shown **(A-H)**.

From triplicate sample analysis, we identified 195 proteins as commonly expressed in both the *E. chaffeensis* DC and RC forms (**Supplementary Table S1**). In addition, 189 proteins were identified as exclusively expressed in the RC form (**Supplementary Table S2**). The DC proteome included approximately 94% of proteins with molecular weights between 10–100 kDa. The LC-MS/MS data identified 60% of proteins as having more than 10% sequence coverage for the DC proteome and, similarly, 68% of proteins were identified with more than two unique peptides (**Supplementary Figure 2A**). Similarly in the RC proteome, 92% of the identified proteins had molecular weights ranging from 10–100 kDa. More than two unique peptides per protein were identified with 73% of the proteins having greater than 10% sequence coverage (**Supplementary Figure 2B**). We assessed the KEGG-based annotations (https://www.genome.jp/kegg/annotation/) for functional assignment distribution of the identified proteins into different categories of biological processes (**Figure 2**). Many of the expressed proteins belonged to proteins with unknown functions: 59 and 144 in DCs and RCs, respectively. Most proteins with known functions in the DC and RC forms belonged to metabolic processes: 96 and 195, respectively. The second most abundant group of proteins with known function belonged to transport (33 in DCs and 82 in RCs, respectively), followed by proteins that belonged to the regulation of biological processes (20 in DCs and 32 in RCs), and immune response category proteins represented the next most abundant group with DC form having more proteins (24 in DCs and 17 in RCs).

### Proteins expressed in *E. chaffeensis* DC and RC forms

Among the total proteins detected, 66 were listed as the most abundant proteins with 10 peptides or more per protein in RC and DC fractions (**Table 1**). They included an ankyrin repeat protein, the outer membrane protein assembly factor (BamA), 60 kDa chaperonin, peptidylprolyl isomerase, DnaK, conserved domain proteins, putative outer membrane protein TolC, an uncharacterized protein, periplasmic serine endoprotease DegP-like (PEN), putative lipoproteins, DNA-binding response regulator (DRR), three p28-Omp proteinsm and a T4SS protein (Virb9). The most abundantly expressed proteins found in the RC form included a putative ribosomal protein S1 (rpsA), bifunctional protein PutA, ClpB, dihydrolipoyl dehydrogenase (lpdA), succinate dehydrogenase flavoprotein subunit (sdhA), transcription termination/antitermination protein (NusA), ATP-dependent zinc metalloprotease (FtsH), malate dehydrogenase (MDH), DNA gyrase subunit B (gyrB), and putative iron-binding protein (IB), among others (**Table 1**).

**Table 1:** The most abundantly expressed proteins in the DC and/or RC forms.

Gene ID	Description	Unique peptides (DC)	Unique peptides (RC)
ECH_0684	Ankyrin repeat protein	33	41
ECH_1071	Outer membrane protein assembly factor (BamA)	19	36
ECH_0365	60 kDa chaperonin	24	30
ECH_0731	Peptidylprolyl isomerase	20	29
ECH_0471	Chaperone protein (DnaK)	19	29
ECH_0525	Conserved domain protein	19	28
ECH_0526	Conserved domain protein	12	22
ECH_1020	Putative outer membrane protein TolC	10	20
ECH_0593	Uncharacterized protein	13	20
ECH_1052	Periplasmic serine endoprotease DegP-like	10	19
ECH_0929	Putative lipoprotein	12	18
ECH_1012	DNA-binding response regulator	13	17
ECH_1005	Outer membrane protein assembly factor BamD	10	17
ECH_1065	Succinyltransferase component of 2-oxoglutarate dehydrogenase complex (sucB)	11	17
ECH_0707	Uncharacterized protein	21	17
ECH_1144	Major outer membrane protein P28-1	13	14
ECH_1126	Major outer membrane protein OMP-1U	10	13
ECH_1124	Major outer membrane protein OMP-1P	12	12
ECH_0043	Type IV secretion system protein VirB9	10	12
ECH_0402	Putative ribosomal protein S1 (rpsA)	9	30
ECH_0667	Bifunctional protein (PutA)	4	26
ECH_0367	Chaperone protein (ClpB)	4	26
ECH_0509	Dihydrolipoyl dehydrogenase (lpdA)	9	23
ECH_0315	Succinate dehydrogenase flavoprotein subunit (sdhA)	4	19
ECH_0562	Transcription termination/antitermination protein (NusA)	6	19
ECH_1098	ATP-dependent zinc metalloprotease (FtsH)	4	19
ECH_0175	Malate dehydrogenase	4	19
ECH_0620	DNA gyrase subunit B (gyrB)	3	19
ECH_0189	Putative iron-binding protein	10	18
ECH_0702	Folylpolyglutamate synthase (folC)	9	18
ECH_0383	Type I secretion system ATPase	1	17
ECH_0862	Uncharacterized protein	4	16
ECH_1050	Protein HflK	5	16
ECH_0970	Type I secretion membrane fusion protein, HlyD family	1	16
ECH_0475	Signal recognition particle protein(ffh)	4	16
ECH_1041	Type IV secretion system protein VirB4	2	15
ECH_0886	Acetylornithine/succinyldiaminopimelate aminotransferase (argD)	8	14
ECH_0558	Putative lipoprotein	5	14
ECH_0042	Type IV secretion system protein VirB10	8	14
ECH_1051	Protein HflC	5	14
ECH_0224	Inosine-5’-monophosphate dehydrogenase	4	14
ECH_0760	RNA polymerase sigma factor (RpoD)	2	14
ECH_1121	Major outer membrane protein Omp-1N	8	13
ECH_0676	Arginine biosynthesis bifunctional protein (ArgJ)	2	13
ECH_0369	Probable cytosol aminopeptidase	8	13
ECH_0782	Membrane protein insertase YidC	5	13
ECH_0997	ATP-dependent protease ATPase subunit (HslU)	2	13
ECH_0734	Antioxidant, AhpC/Tsa family	6	12
ECH_1072	Outer membrane protein, OmpH family	9	12
ECH_1123	Major outer membrane protein OMP-1Q	6	12
ECH_1133	Major outer membrane protein OMP-1H	9	12
ECH_0058	2,3,4,5-tetrahydropyridine-2,6-dicarboxylate N-succinyltransferase(dapD)	8	12
ECH_0048	TPR domain protein	5	12
ECH_0044	VirB8	3	11
ECH_0293	Disulfide oxidoreductase	9	11
ECH_1129	Major outer membrane protein OMP-1W	9	11
ECH_0128	Putative lipoprotein	2	11
ECH_0900	ClpX	3	11
ECH_0097	Fructose-biphosphate aldolase (fba)	4	11
ECH_0041	Type IV secretion system protein VirB4	2	11
ECH_1006	Phosphoribosylamine–glycine ligase(purD)	2	11
ECH_0210	SurA domain protein	2	11
ECH_0339	Putative nitrogen regulation protein NtrX	2	11
ECH_0976	Major antigenic protein	7	10

In total, 189 proteins expressed primarily in the RC form were found to be in increasing abundance (**Supplementary Table S2**). Of these, IB, UvrABC system protein A (uvrA), two T4SS proteins (VirB4 and VirB11), 2-oxoglutarate dehydrogenase (sucB), and putative response regulator/diguanylate cyclase (pleD) showed higher abundance of expression. Among the abundantly expressed proteins that are involved in DNA replication and cell division are FtsK and FtsA, DNA helicase, DNA polymerase III subunit gamma/tau (dnaX), DNA polymerase I, transcription elongation factor (GreA), ribosome-binding ATPase (YchF), replicative DNA helicase (dnaB), DNA topoisomerase 1(topA), RNA polymerase beta-prime subunit (rpoC), and prolyl-tRNA synthetase (ProRS). Furthermore, several proteins involved in cellular transport function in the RC fraction included efflux transporter, phosphate ABC transporter and permease/ATP-binding protein (PAP), ATP-binding protein (AP), and T4SS proteins, such as VirB6 and VirB8.

### Upregulated expression of proteins in the RC form of *E. chaffeensis*

**Figure 3:**
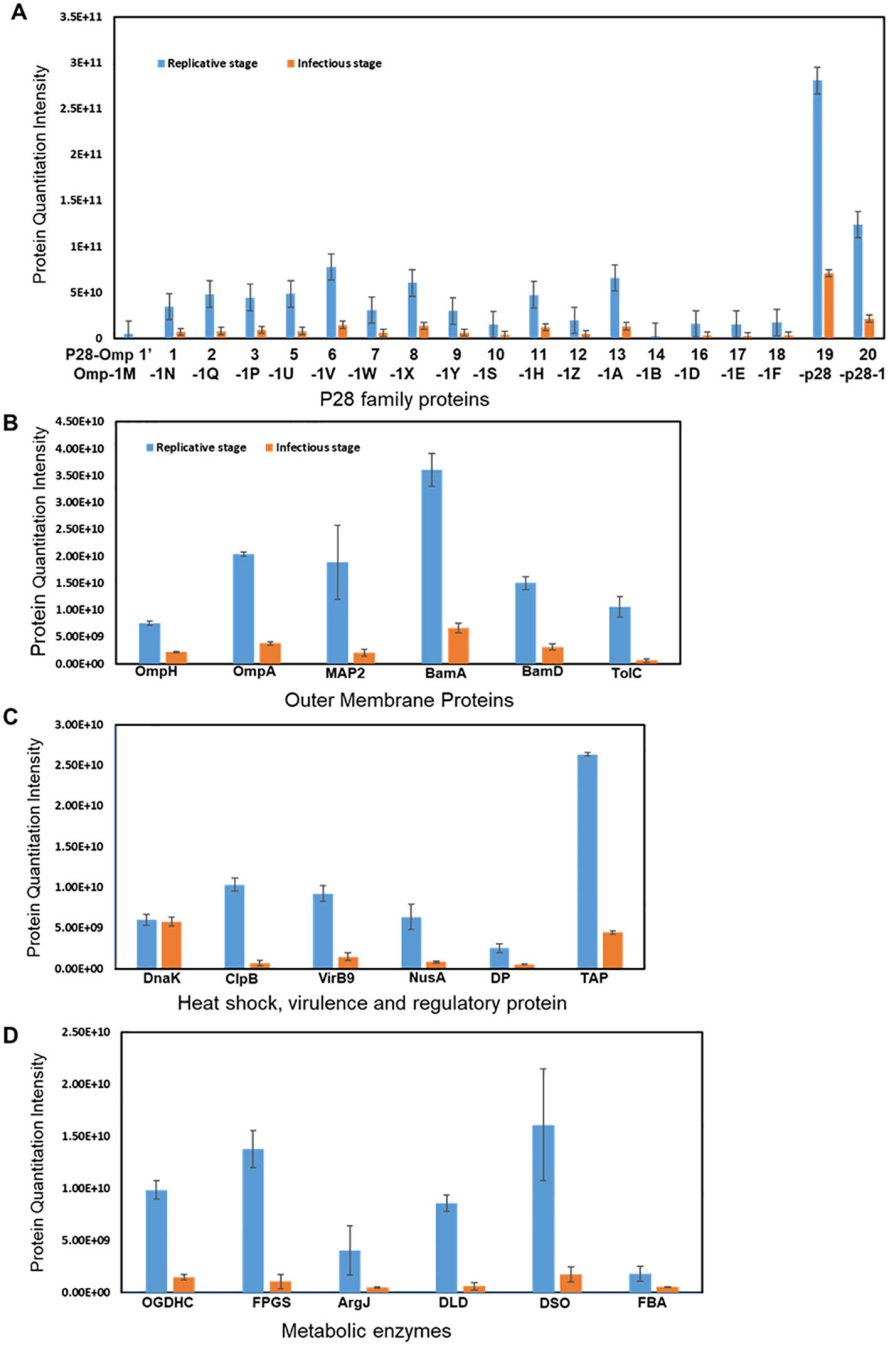
Differential expression of proteins in the RC and DC forms of *E. chaffeensis*. Increased abundance of p28 family proteins **(A)**; outer membrane proteins **(B)**; heat shock, virulence, and regulatory protein **(C)**; and metabolic enzymes **(D)** in the replicative growth form (RC>DC).

**Figure 4:**
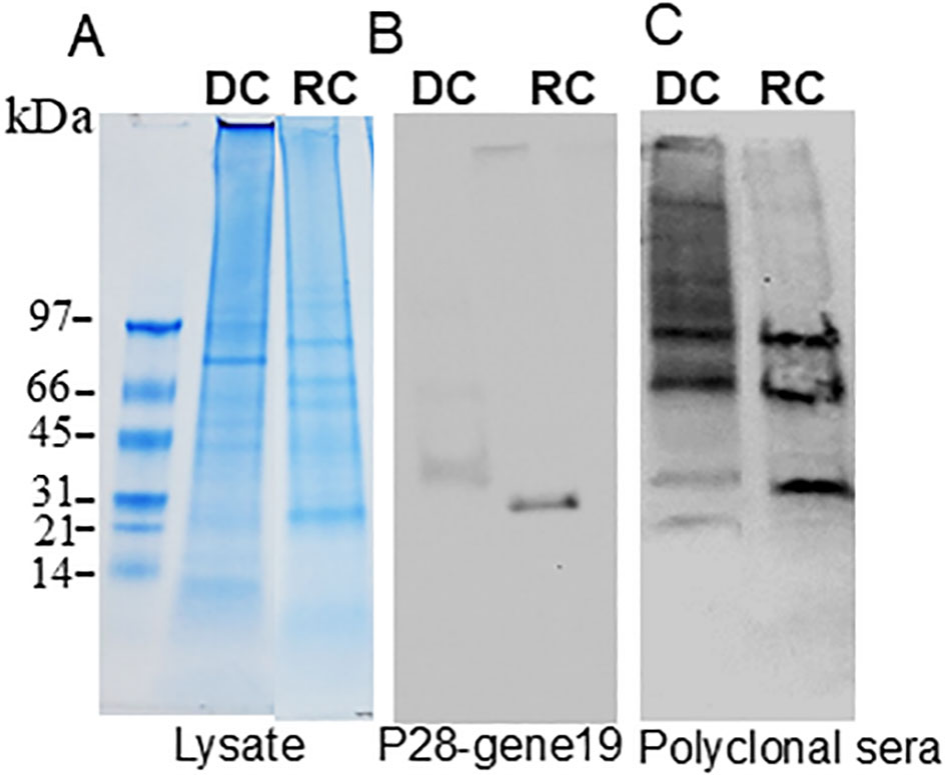
Immunoblotting of *E. chaffeensis* DC (replicate 1) and RC (replicate 1) proteins. In total, 20 μg of proteins from each form were separated on 4%–20% SDS-PAGE gel **(A)**, and the resolved proteins were transferred to nitrocellulose membranes and immunoblotted with p28–gene19 monoclonal antibody **(B)** or with *E. chaffeensis* polyclonal sera **(C)**.

Protein expression was determined by comparing the MS1 intensity values calculated by dividing a protein’s total intensity by the number of tryptic peptides from the three replicate samples of RCs and DCs. Expression changes were considered significant with a p-value < 0.05 and a false discovery rate (FDR) <1%. Based on these criteria, proteins associated with the p28-Omp gene family were predominantly upregulated in the RC form compared to the DC form (**Figure 3A**). Among the expressed p28-outer membrane proteins, p28-Omp 19 had the highest expression, followed by p28-Omp 20 and p28-Omp 6, respectively. BamA, BamD, OmpA, OmpH, and TolD were also among the highly expressed proteins in the RC form (**Figure 3B**). Stress response and virulence-associated proteins, such as ClpB, transcription activator proteins (TAP), VirB9, and transcriptional regulation proteins were also upregulated in the RC form (**Figure 3C**). Metabolic enzymes such as 2-oxoglutarate dehydrogenase complex (OGDHc), folylpolyglutamate synthase (FPGS), arginine biosynthesis bifunctional protein (ArgJ), dihydrolipoyl dehydrogenase (DLD), disulfide oxidoreductase (DSO), and fructose-biphosphate aldolase (FBA) were found to be abundantly expressed in RCs compared to DCs (**Figure 3D**). To confirm the MS-based quantitative protein expression data, p28-Omp 19 abundant expression in RCs was also confirmed by 1D Western blot analysis using p28-Omp 19-specific monoclonal antibody and *E. chaffeensis* polyclonal sera, respectively (**Figure 4**).

## Discussion

The developmental cycle of *E*. *chaffeensis* involves the infectious DC form and the non-infectious RC form. We hypothesized that unique protein composition exists in these two forms to support bacterial active replication in phagosomes and, in the less active and smaller-sized DC form, to support its release and infect naïve host cells. In our previous studies, we cataloged the transcriptomes and proteomes in *E. chaffeensis* and how mutations at certain genomic locations impact the protein/gene expressions (Kondethimmanahalli and Ganta, 2018; Kondethimmanahalli et al., 2019). Similarly, we reported specific differences in the RC and DC forms by transmission electron microscopy (DeDonder et al., 2012). As part of our previous studies, we standardized methods for purifying the *E*. *chaffeensis* DC and RC forms from infected host cells which involved the use of density gradient centrifugation (Kondethimmanahalli and Ganta, 2018; Eedunuri et al., 2018; Kondethimmanahalli et al., 2019; Zhang et al., 2021). In the current study, isolated DC and RC form proteome compositions were investigated. Prior studies by confocal microscopy analysis suggested that the p28 outer membrane proteins (p28) are highly expressed in the RC form, while an envelope glycoprotein GP120 (gp120) was a cellular marker specific to the DC form (Zhang et al., 2007). A 47kDa glycoprotein is another DC-specific protein (Doyle et al., 2005a). The EtpE surface protein that facilitates adhesion and entry of *E. chaffeensis* into mammalian cells was found to be upregulated in DCs (Moumène and Mayer, 2016).

The proteome analysis using high-resolution LC-MS/MS aided in obtaining comprehensive detection and quantitation of *E. chaffeensis* expressed proteins. The LC-MS/MS data for DCs and RCs, assessed using scatter plot analysis, revealed significant correlation among replicate samples of each form, but not when comparing the DC form with the RC form. Most of the proteins were identified with two or more peptides and had high sequence coverage spanning more than 10%. The identified peptide Xcorr values, a measure of experimental peptide fragments to the theoretical spectra, were above the specified score threshold (>2.0) (Klammer et al., 2009).

We report twice the number of expressed proteins in the metabolically active RC form compared to the compact and less active DC form, which is also approximately half the size of RC form. While having more expressed proteins in the RC form is anticipated, the detection of nearly identical commonly expressed proteins in both DC and RC forms is novel. We previously reported metabolic activity with active protein synthesis occurring primarily in the RC form (Eedunuri et al., 2018).

There was abundant expression of the ankyrin repeat protein, chaperone proteins, DNA binding response regulator (DRR), a peptidylprolyl isomerase (PPI), and conserved domain proteins in both DC and RC forms as they are likely housekeeping proteins essential for establishing the infection and replication. In previous global transcriptome and proteome studies by us and others, similar trends of the abundance of expressed proteins are reported (Lin et al., 2011; Kondethimmanahalli and Ganta, 2018; Kondethimmanahalli et al., 2019) suggesting that these proteins are ubiquitously expressed independent of the infectious and replicative nature. Several proteins, such as rpsA, PutA, ClpB, lpdA, sdhA, NusA, FtsH, gyrB, IB, type I secretion system ATPase (T1SS ATPase), type I secretion membrane fusion protein, (T1SS fusion protein), OMPs, and T4SS proteins, were among the most highly expressed proteins in the RC form, indicating that replication and transition from DCs to RCs require energy-associated pathways and metabolic machinery to synthesize proteins associated with cell envelope function, metabolism, and cellular transport (Skipp et al., 2016). Similarly, prior total quantitative proteome analyses revealed that chaperones, enzymes involved in biosynthesis and metabolism, and outer membrane proteins essential for survival and pathogenesis are highly expressed in *E*. *chaffeensis (* Lin et al., 2011). These proteins were also identified as highly expressed proteins in the RC form described in the current study. We speculate that maintaining the protein synthetic machinery is crucial during the transition process and hence these proteins are highly expressed in the RC form. The proteins associated with metabolic processes, transport, regulation of biological processes, and response to stimulus were represented abundantly in the RC form compared to the DC form. Indeed, pronounced changes with a higher expression of proteins involved in bacterial transport and DNA and protein biosynthesis indicate that a large amount of energy is invested in the protein synthesis belonging to different biological processes during the transition from the DC to RC form (Skipp et al., 2016). In contrast, we detected more expressed immune response proteins in the DC form that support host interactions by defining the immune response in a new infection cycle (Mukhopadhyay et al., 2006).

The protein levels of p28-Omps, ClpB, T4SS-VirB, transcriptional activators, and metabolic enzymes were generally higher in RCs. The upregulation of the complete set of p28-Omps in the RC form strongly indicates that *E. chaffeensis* expends significant energy in synthesizing proteins associated with cell envelope function. The p28-Omp multigene locus includes 22 genes encoding for the bacterial immunogenic membrane proteins essential for immune response and adhesion functions in *E. chaffeensis* and other closely related species (Ohashi et al., 1998; Reddy et al., 1998; Yu et al., 2000; Kumagai et al., 2006; Singu et al., 2006; Seo et al., 2008). Consistent with previous studies, p28-Omps are among the most abundantly expressed immunogenic proteins. Omp-p28 (also known as p28-Omp 19) is the most abundantly expressed protein from this p28-Omp multigene locus and this result is consistent with previous reporting of its transcript in macrophage culture-grown *E. chaffeensis* (Kondethimmanahalli et al., 2018 and 2019). Previous reports showed that P28-Omp multigene family proteins have been detected by proteomic methods in *E. chaffeensis* infected with HL-60 cells (Lin et al., 2011).

*E. chaffeensis* encodes several genes for chaperones involved in cell homeostasis and oxidative stress response (Felek et al., 2003; Sexton and Vogel, 2002; Ohashi et al., 2002). Our study has generated quantitative data for DnaK and ClpB. The presence of such proteins is critical for *E*. *chaffeensis* replication and stress response (Kuczynska-Wisnik et al., 2017) and differential expression of these proteins observed in the current study is consistent with the prior documentation of mRNA expression for these proteins (Zhang et al., 2013). Virulence-associated proteins, such as VirB9, are involved in undermining the host immune response (Green and Mecsas, 2016; Rapisarda and Fronzes, 2017) thereby contributing to the pathogenicity in *E. chaffeensis* and other rickettsiales (Rikihisa et al., 2009; Lopez et al., 2007; Felek et al., 2003). In general, pathogenic bacteria use metabolic enzymes to generate energy in the form of ATP (Eisenreich et al., 2013; Olive and Sassetti, 2016). The abundant synthesis of these metabolism enzymes in the RC form may increase the metabolic activity to maintain a protein synthetic capability required for extracellular survival.

## Conclusions

We report that the protein profiles of the RC and DC forms were very distinct, reflecting the specific functional priorities of the two morphological forms for establishing infection and cellular differentiation in host cells. The RC form expressed two-fold more proteins than the DC form, suggesting increased protein synthesis and metabolic activity during the replicative period. The RC and DC form-specific proteomic data provide insights into the role of protein expression dynamics of *E. chaffeensis*, thus serving as a fundamental resource for the development of novel diagnostics or vaccine candidates targeting either the infectious or replicative stages of the pathogen.

## Data Availability

The data presented in the study were deposited in the Mass Spectrometry Interactive Virtual Environment (MassIVE) repository and accession number is MSV000097332, which can be accessed at https://massive.ucsd.edu/ProteoSAFe.

## Glossary

DC: infectious dense-core cells
RC: non-infectious replicating cells
HME: human monocytic ehrlichiosis
OMP: p28-outer membrane proteins
T4SS: Type IV secretion system
TRP: tandem repeat proteins
Anks: ankyrin proteins
2-DE: two-dimensional electrophoresis
PBS: phosphate buffered saline
DDA: data-dependent acquisition
ClpB: ATP-dependent chaperone
DnaK: chaperone protein
BamA: outer membrane protein assembly factor
TolC: putative outer membrane protein, PEN, periplasmic serine endoprotease DegP-like
DRR: DNA-binding response regulator
rpsA: putative ribosomal protein S1
PPI: peptidylprolyl isomerase
lpdA: dihydrolipoyl dehydrogenase
sdhA: succinate dehydrogenase flavoprotein subunit
NusA: transcription termination/antitermination protein
FtsH: ATP-dependent zinc metalloprotease
gyrB: DNA gyrase subunit B
MDH: malate dehydrogenase, putative iron-binding protein (IB), uvrA, UvrABC system protein A
pleD: putative response regulator/diguanylate cyclase
FtsK: putative cell division protein
dnaX: DNA polymerase III subunit gamma/tau
GreA: transcription elongation factor, YchF, ribosome-binding ATPase, replicative
dnaB: DNA helicase
topA: DNA topoisomerase 1
rpoC: RNA polymerase beta-prime subunit, ProRS, prolyl-tRNA synthetase
PAP: permease/ATP-binding protein
AP: ATP-binding protein
MAP: major antigenic protein
DP: DNA-binding protein
TAP: transcription activator proteins
NusA: transcription termination proteins
OGDHc: 2-oxoglutarate dehydrogenase complex
FPGS: folylpolyglutamate synthase
ArgJ: Arginine biosynthesis bifunctional protein
DLD: dihydrolipoyl dehydrogenase
DSO: disulfide oxidoreductase
FBA: fructose-biphosphate aldolase
T1SS ATPase: Type I secretion system ATPase
T1SS fusion protein: Type I secretion membrane fusion protein.
