# Supplementary figures and images for: Ehrlichia chaffeensis proteomic profiling reveals distinct expression patterns of infectious and replicating forms
Source: Front Cell Infect Microbiol. 2025 Apr 14;15:1463479. doi: 10.3389/fcimb.2025.1463479 (PMC12053472; doi:10.3389/fcimb.2025.1463479)

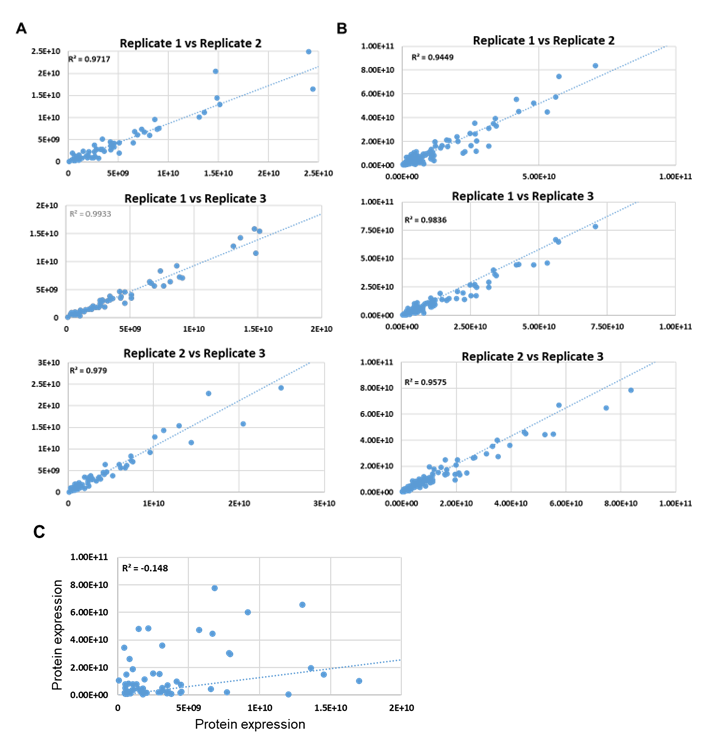

Supplement: Supplementary file 1 [file Image1.tif]
