# Supplementary material for: Ehrlichia chaffeensis proteomic profiling reveals distinct expression patterns of infectious and replicating forms
Source: Front Cell Infect Microbiol. 2025 Apr 14;15:1463479. doi: 10.3389/fcimb.2025.1463479 (PMC12053472; doi:10.3389/fcimb.2025.1463479)
Supplement: Supplementary file 2 [file Presentation2.pptx]

## Slide 1
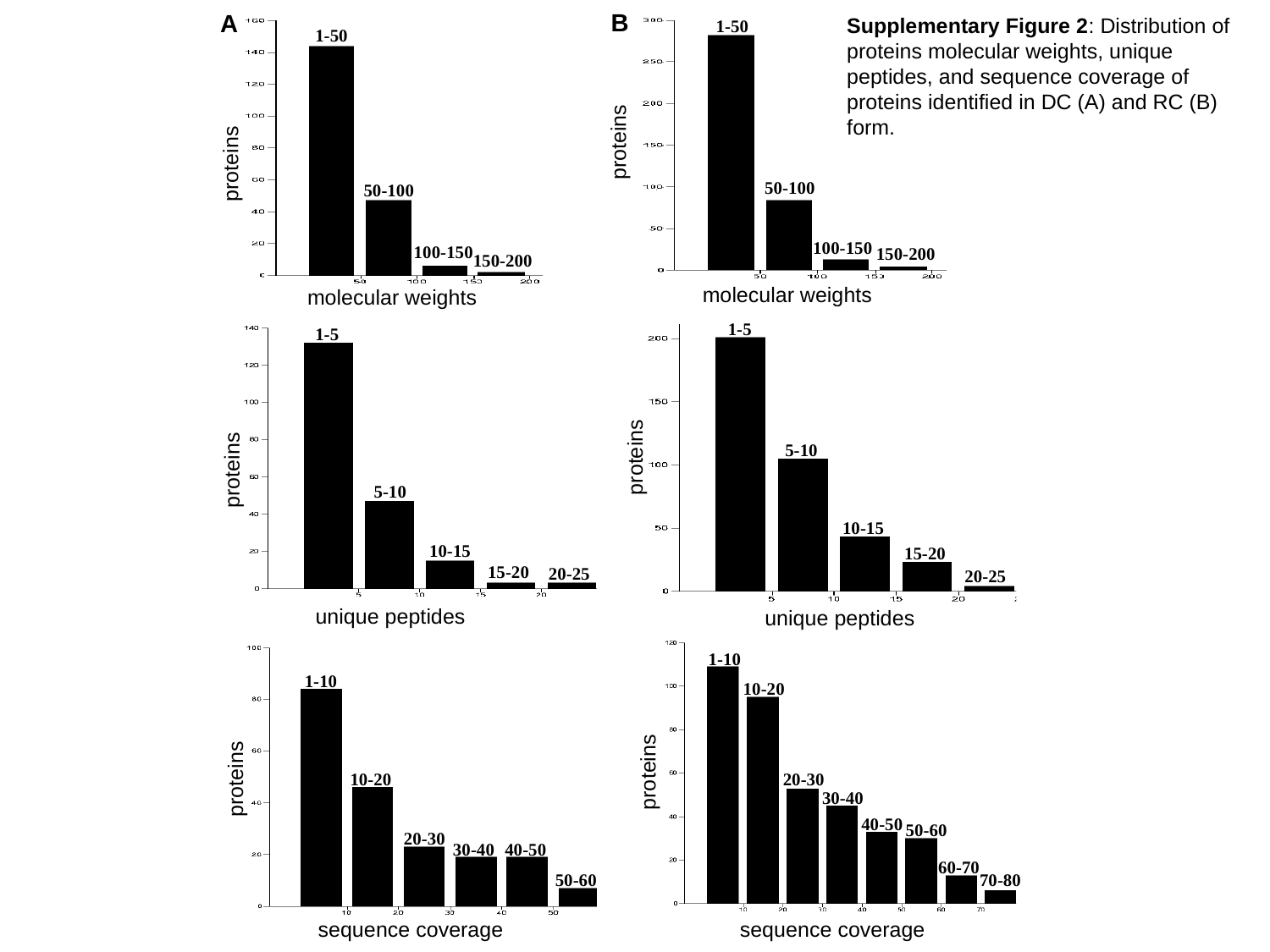

B
A
proteins
proteins
molecular weights
molecular weights
proteins
proteins
unique peptides
unique peptides
proteins
proteins
sequence coverage
sequence coverage
1-50
1-50
50-100
50-100
100-150
100-150
150-200
150-200
1-5
1-5
5-10
5-10
10-15
10-15
15-20
15-20
20-25
20-25
1-10
1-10
10-20
20-30
10-20
30-40
40-50
50-60
20-30
40-50
30-40
60-70
50-60
70-80
Supplementary Figure 2: Distribution of proteins molecular weights, unique peptides, and sequence coverage of proteins identified in DC (A) and RC (B) form.
